# Cardiovascular health and the menopausal woman: the role of estrogen and when to begin and end hormone treatment

**DOI:** 10.12688/f1000research.15548.1

**Published:** 2019-09-03

**Authors:** Frederick Naftolin, Jenna Friedenthal, Richard Nachtigall, Lila Nachtigall

**Affiliations:** 1 Interdisciplinary Program in Menopausal Medicine, Department of Obstetrics and Gynecology, New York University School of Medicine, New York, NY, USA

**Keywords:** estrogen, estradiol, menopause, heart disease, cardioprotection, Timing Hypothesis

## Abstract

Reports have correlated the use of estrogen for the treatment of menopausal symptoms with beneficial effects on the cardiovascular system. Molecular, biochemical, preclinical, and clinical studies have furnished a wealth of evidence in support of this outcome of estrogen action. The prospective randomized Women’s Health Initiative (WHI) and the Early Versus Late Intervention Trial (ELITE) showed that starting menopausal hormone treatment (MHT) within 5 to 10 years of menopause is fundamental to the success of estrogen’s cardioprotection in post-menopausal women without adverse effects. Age stratification of the WHI data has shown that starting hormone treatment within the first decade after menopause is both safe and effective, and the long-term WHI follow-up studies are supportive of cardioprotection. This is especially true in estrogen-treated women who underwent surgical menopause. A critique of the WHI and other relevant studies is presented, supporting that the timely use of estrogens protects against age- and hormone-related cardiovascular complications. Salutary long-term hormone treatment for menopausal symptoms and prevention of complications has been widely reported, but there are no prospective trials defining the correct length to continue MHT. At present, women undergoing premature menopause receive estrogen treatment (ET) until evidence of hormone-related complications intervenes. Normal women started on MHT who receive treatment for decades without hormone-related complications have been reported, and the WHI follow-up studies are promising of long-term post-treatment cardioprotection. A prevention-based holistic approach is proposed for timely and continuing MHT/ET administration as part of the general management of the menopausal woman. But this should be undertaken only with scheduled, annual patient visits including evaluations of cardiovascular status. Because of the continued occurrence of reproductive cancers well into older ages, these visits should include genital and breast cancer screening.

## Introduction

The age-related cessation of ovarian follicle development and surgical removal of the ovaries are followed by dramatic declines of circulating estrogens, especially estradiol. Depending on the individual woman, the decline of circulating estrogen is followed by numerous signs and symptoms of estrogen deficiency: vasomotor episodes, sleep disorders, depression, metabolic imbalance, diminished bone mineral mass, and decreased skin turgor, to enumerate the most well-known symptoms and complications of menopause ^1^. These effects of decreased estrogen are commonly and successfully treated with menopausal hormone treatment (MHT): estrogen alone for women who have had their ovaries and uterus removed (estrogen treatment, or ET) or estrogen plus a progestin for those whose endometrium remains. The results are generally very prompt and satisfactory.

If the MHT is begun within the first few years after menopause, the benefits far outweigh the risks for most women. This has been shown to be an effective pharmacological approach to post menopausal health ^2^. This review concerns an associated and controversial issue in women’s health: the possible cardioprotective value of estrogen administration in menopausal women and how the lessons learned in testing for this additional positive effect of MHT have better delineated prevention as a lifelong approach to women’s wellness. It does not address the management of established cardiac disease, such as cardiac failure.

As will be seen, although the evidence favors cardioprotective effects of timely estrogen administration of the post-menopausal woman, the final proof—prospective evidence of reduced cardiac events—remains to be obtained. Without such final proof, the inclusion of cardioprevention as the justification for MHT is not warranted. Rather, it is prudent to develop an approach to the management of the post-menopausal woman that is founded on patient education, lifestyle modification, and treatment that includes MHT/ET in a way that offers the possible added value of cardioprotection without conferring the additional burden of unwanted side effects ^3^.

## Estrogen and cardioprotection

**Figure 1.**
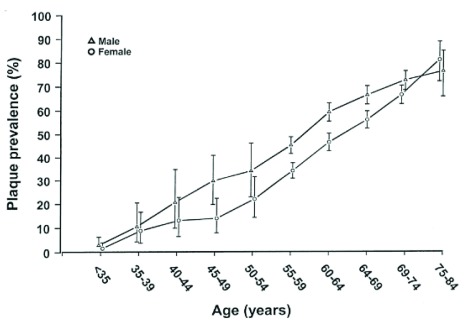
Prevalence of carotid atherosclerosis by age and sex. Reprinted from Joakimsen *et al*. ^14^ with permission.

For more than 50 years, estrogen has been considered cardioprotective. The first suggestions arose from sex differences in clinical presentation of myocardial symptoms and events; angina and infarction appear about a decade earlier in men than in women ^4, 5^. Since women’s cardiac events usually followed the cessation of ovarian function (natural or induced menopause) ( Figure 1), it has been proposed that the loss of estrogen was causal and that the roughly 10-year hiatus reflected the effects on the cardiovascular system of the loss of estrogen-secreting ovarian follicles ^6^. Epidemiological studies have supported this idea ^7^.

There are myriad proposed mechanisms by which endogenous estrogen may protect against cardiovascular disease (CVD). One proposed mechanism is that estrogen administration has a known positive impact on plasma lipid profiles, anti-platelet effects, and anti-oxidant effects ^8– 10^. Previous studies have shown that post-menopausal women experience greater arterial stiffening, a known marker of vascular aging, than pre-menopausal women ^11^, and a study by Moreau *et al*. suggests that estrogen improves endothelial-dependent vasodilatation ^12^.

Estrogen receptors and aromatase are present in human coronary artery endothelium ^13^. Estrogen receptors have profound action on muscles and insulin action, both of which are necessary for maintenance of vessels ^15^. Abnormalities of estrogen receptor alpha have been linked to CVD ^16^. Abnormalities of aromatase are rare, probably due to the requirement for aromatase during reproduction, and have been associated with early atherogenesis ^17, 18^.

Estradiol has also been shown to inhibit monocyte adhesion to vascular endothelium, a known step in the development of atheromas and arteriosclerosis ^19^. Evidence now suggests that this process is mediated by sex steroid–induced inhibition (more specifically, sialylation) of neural cell adhesion molecules (nCAMs), which are among the early arresting mechanisms for capturing leukocytes in areas of inflammation; sex steroid–induced sialylation of nCAM molecules prevented attachment of monocytes to endothelial cells ^20^. Similar effects on leukocyte capture by hormonal manipulation, but not directly related to cardioprotection have been published ^21, 22^.

## Many clinical studies support the cardioprotective effects of estrogen treatment on the incidence/progression of cardiovascular disease in estrogen-deficient women

Contrary to the prevalent understanding among patients and clinicians, the Women’s Health Initiative (WHI) has shown that when administered to menopausal women between 50 and 54 years of age, ET is clearly cardioprotective ^23^. However, the roll-out of this data over the years has been confusing (see below).

On the contrary, the Nurses’ Health Study (NHS), one of the largest ongoing observational studies of women (59,337 ongoing subjects), has been very clear. In its latest review of cardioprotection the NHS continued to show a highly significant decrease in the relative risk of major coronary disease—odds ratio 0.45, confidence interval (CI) 0.34–0.60—in women who received MHT ^24, 25^. Langer’s ^26^ review of the results of 10 observational studies showed that, in all studies except one, MHT reduced the risk of CVD, and several studies showed marked reduction on cardiovascular risk ^27, 28^. Other publications based on reviews of recent observational studies, including the effect of menopausal age or MHT on heart failure, have not shown a definitive role of estrogen, nor of MHT, in outcomes ^29, 30^.

A large Bayesian analysis by Salpeter *et al*. included both premature and natural menopausal women ^7^. It makes a strong case for estrogen protection against cardiovascular complications, especially among post-ovariectomy women, in those who receive estrogen immediately following surgical menopause ^7^. In addition, the same group has shown the cost-effectiveness of ET in young menopausal women taking estrogen for 5 to 30 years ^31^. The first retrospective study of both carotid intima-media thickness (CIMT) and coronary artery calcium (CAC) showed that post-menopausal women taking MHT had statistically significant protection compared with non-treated controls ^32^.

In addition to pioneering epidemiological studies supporting estrogen, Hodis, Mack and Lobo have shown, in the prospective randomized Early Versus Late Intervention Trial (ELITE), that ET inhibits the progress of imaging markers of CVD ^33^ (see below). Aspects of the effects of MHT, including the role of the progestins, other than cardiovascular effects were reviewed by Lobo *et al*. ^33^ and will not be covered in this contribution.

## Two large randomized clinical trials initially furnished negative results that have been corrected by improved interpretation and further research

The WHI’s initial repudiation of the cardioprotective effects of estrogen has driven the confirmation of the beneficial use and safety of both ET and MHT and furnished evidence of the importance of the timing of the start of MHT.

Over the past two decades, the cardioprotective effects of estrogen have been questioned, largely by the interpretation of the results of two large, National Institutes of Health–sponsored randomized clinical trials: the Heart and Estrogen/Progestin Replacement Study (HERS) ^34^ and the WHI ^35^. Both studies showed increased cardiovascular events during the first year of hormone treatment (HT). These studies drew great attention because of their large numbers of subjects and their report of early harm (increased venous thromboembolism, or VTE) from the use of MHT/ET ^36^. In fact, the adverse outcomes in the first years of the HERS and WHI appear to be traceable to the higher incidence of estrogen-induced VTE in subjects at higher risk than the normal population ^37^. We and others have addressed the causes of these unexpected findings and why the unstratified for age report of the WHI ^35^ did not represent findings in women who received treatment for early post-menopausal symptoms ^38, 39^. To be more precise, in the HERS trial of what was termed “secondary cardio-prevention by estrogen”, all subjects had active clinical CVD (myocardial infarctions/intractable angina pectoris) and were prone to VTE ^35^. The subjects received continuous combined conjugated equine estrogen (CEE) and medroxyprogesterone acetate (MPA). Controls received placebo pills. Although it reduces liver production of lipids, oral estrogen also induces thrombogenic liver proteins. The balance of salutary (lipid-lowering) versus harmful (thrombogenic) effects favors the latter in subjects with endothelial disease, as is found in women who are well past menopause or have disordered vascular endothelium or both ^36^. In the HERS, the average age of the women who experienced previous cardiovascular events and began administration of estrogen plus MPA was 66.7 years. Therefore, all HERS subjects were at high risk for both VTE and its complications or rapid recurrence of their underlying CVD. Not surprisingly, VTE, stroke, and recurrence of cardiovascular events appeared in the first year of the treatment, which stopped the HERS. Additional issues are mentioned in the following paragraphs.

In the WHI trial, the women in the two HT arms received daily oral CEE plus MPA (intact, with natural menopause) or daily oral CEE (premature menopause, post-hysterectomy). Both groups of HT began MHT at an average of about 63 years of age and were more than 10 years post-menopause when starting treatment. When the results were examined without stratification for age, it was found that there was an excess of VTEs in the first year of the trial ** in the women who received treatment ** compared with placebo subjects ^37^. Since the subjects in the WHI had risk factors for CVD but no demonstrated clinical CVD, the cause of the excess VTEs has undergone considerable thought and investigation. Stratification of the WHI into age groups and re-analysis of the outcomes showed that the cardiovascular complications are limited mainly to women who began MHT after a decade of absent ovarian estrogens ^38^.

## Comparison of the WHI subjects with the usual perimenopausal woman

Since the age and composition of the full WHI cohort made it impossible to evaluate MHT rather than HT ^39^, it is important to understand how the WHI cohort was constructed. The average age of women in the US at the time of menopause is 51.4 years. Those treated receive MHT for symptoms, mainly hot flushes. In contradistinction, the WHI tested older women and not more than 10% were symptomatic. Since the typical woman begins to decrease her vasomotor symptoms at 5 years and they are usually gone by 7 years past menopause ^40^, the WHI tested HT and not MHT. The reasons for the inclusion of women of such advanced ages are as follows: (1) Because the effect of estrogen on hot flushes would differentiate between treatment and placebo groups and this could affect adherence to the assigned treatment protocol, moderately symptomatic women were limited to 10% in the WHI; this required inclusion of older, non-symptomatic, post-menopausal women. (2) Since the end points used in the WHI were to be actual cardiovascular events, such as myocardial infarction and intractable angina, and the expected rate of these end points in newly menopausal women during the proposed 7-year length of the study was very low, the power of the WHI to show significant differences between treatment and placebo groups would require prohibitive numbers of study subjects. To satisfy the need to restrict the number of symptomatic subjects and to include subjects with higher risk, it was decided to include women distant from menopause ^40^. That is, the WHI subjects were as old as 79 years (average of about 63 years) when they started hormones ^36^. Since most of the subjects were more than 10 years past menopause and were not symptomatic, these women were receiving hormone treatment not resembling MHT for the menopause.

## Analysis of the factors leading to excess adverse cardiovascular events in the WHI

After the application of sub-analyses and additional study designs, the WHI has furnished more useful insights into the role of hormones in cardioprotection and age-related CVD. Re-analysis of the WHI subjects for VTE and effects of HT showed that the excess of the main complication, VTE, occurred mainly in the first year of treatment and among the more elderly subjects ^41^. Thus, sub-clinical CVD with endothelial deterioration may have been betrayed by the induction of thrombogenic liver proteins inherent in the hormonal agents used in the WHI. The younger women in the WHI cohort, having normal vascular endothelium, would not have suffered VTE. A similar situation occurred in the HERS study, in which women with recent CVD events had recurrence in the first year of treatment with hormones ^34^. Further analysis of the WHI outcomes is available ^42^. However, the analysis of the effect of the constant dosing regimen of the WHI versus cyclic regimens in other, positive studies has not been reported. Similarly, in none of the recently reported studies has there been a dose-response protocol.

## WHI sub-group analyses and imaging studies show that estrogen treatment is cardioprotective

Sub-group analyses of the WHI have explained the discrepancy between the WHI and so many previous studies. The main finding was the breakdown according to age and to estrogen-plus-progestin versus ET. Among subjects who had previously undergone hysterectomy and were 50 to 59 years old at the start of the study, ET women had lower coronary heart disease risk during and following CEE ^43^. The 50- to 54-year-olds, who were more akin to normal menopausal women who received treatment with MHT, had an even more striking improvement of cardiovascular markers ^43^. In 2007, Rossouw *et al*. performed a secondary analysis of the WHI trials, paying attention to age and years since menopause ^44^. Their results showed a trend toward reduced CVD risk in women who started hormone therapy at a younger age or closer to menopause or both ( Table 1 and Table 2) ^44^. Whereas MHT with ET is clearly effective, MHT with MPA is not. Because MPA is a progestin and reduces expression of estrogen receptors, the difference in outcomes between the two treatments may be ascribed to the progestin ^45– 47^.

**Table 1.** Cardiovascular and global index events by age at baseline. Reprinted from Rossouw *et al*. ^44^ with permission.

	Age Group at Randomization									*P* Value for Trend †
	50–59y			60–69y			70–79y			
	No. of Cases			No. of Cases			No. of Cases			
	Hormone Therapy (n=4476)	Placebo (n=4356)	HR (95% CI) *	Hormone Therapy (n=6240)	Placebo (n=6122)	HR (95% CI) *	Hormone Therapy (n=3100)	Placebo (n=3053)	HR (95% CI) *	
**Combined Trials**										
CHD ‡	59	61	0.93 (0.65-1.33)	174	178	0.98 (0.79-1.21)	163	131	1.26 (1.00-1.59)	.16
Stroke	44	37	1.13 (0.73-1.76)	156	102	1.50 (1.17-1.92)	127	100	1.21 (0.93-1.58)	.97
Total mortality	69	95	0.70 (0.51-0.96)	240	225	1.05 (0.87-1.26)	237	208	1.14 (0.94-1.58)	.06
Global index §	278	278	0.96 (0.81-1.14)	717	661	1.08 (0.97-1.20)	606	528	1.14 (1.02-1.29)	.09
**CEE Trial**										
	**CEE** **(n=1637)**	**Placebo** **(n=1673)**		**CEE** **(n=2387)**	**Placebo** **(n=2465)**		**CEE** **(n=1286)**	**Placebo** **(n=1291)**		
CHD ‡	21	34	0.63 (0.36-1.09)	96	106	0.94 (0.71-1.24)	84	77	1.13 (0.82-1.54)	.12
Stroke	18	21	0.89 (0.47-1.69)	84	54	1.62 (1.15-2.27)	66	52	1.21 (0.84-1.75)	.62
Total mortality	34	48	0.71 (0.46-1.11)	129	131	1.02 (0.80-1.30)	134	113	1.20 (0.93-1.55)	.18
Global index §	114	140	0.82 (0.64-1.05)	333	342	1.01 (0.86-1.17)	300	262	1.16 (0.98-1.37)	.01
**CEE + MPA Trial**										
	**CEE + MPA** **(n=2839)**	**Placebo** **(n=2683)**		**CEE + MPA** **(n=3853)**	**Placebo** **(n=3657)**		**CEE + MPA** **(n=1814)**	**Placebo** **(n=1762)**		
CHD ‡	38	27	1.29 (0.79-2.12)	78	72	1.03 (0.74-1.43)	79	54	1.48 (1.04-2.11)	.70
Stroke	26	16	1.41 (0.75-2.65)	72	48	1.37 (0.95-1.97)	61	48	1.21 (0.82-1.78)	.56
Total mortality	35	47	0.69 (0.44-1.07)	111	94	1.09 (0.83-1.44)	103	95	1.06 (0.80-1.41)	.19
Global index §	164	138	1.10 (0.87-1.38)	384	319	1.15 (0.99-1.34)	306	266	1.13 (0.95-1.33)	.96

Abbreviations: CEE, conjugated equine estrogens; CHD, coronary heart disease; CI, confidence interval; HR, hazard ratio; MPA, medroxyprogesterone acetate. *Cox regression models stratified according to prior cardiovascular disease and randornization status in the Dietary modification Trial. †Test for trend (interaction) using age as continuous (linear) form of categorical coded values. Cox regression models stratified according to active vs placebo and trial, including terms for age and the interaction between trials and age. ‡Defined as CHD death, nonfatal myocardial infarcation, or definite silent myocardial infarction (Novacode 5.1 or 5.2). §Defined as CHD, stroke, pulmonary embolism, breast cancer, colorectal cancer, endometrial cancer for CEE plus MPA trial only, hip fracture, or death from other causes.

**Table 2.** Estimated absolute excess risk per 10,000 person-years by age group at baseline. Reprinted from Rossouw *et al*. ^44^ with permission.

	Combined Trials			CEE Trial			CEE + MPA Trial		
	Cases per 100 Person-Years			Cases per 100 Person-Years			Cases per 100 Person-Years		
	Hormone therapy	Placebo		CEE	Placebo		CEE+MPA	Placebo	
CHD Age Group, y 50–59 60–69 70–79	0.20 0.46 0.90	0.22 0.48 0.72	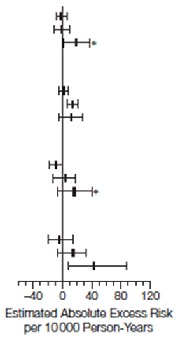	0.17 0.58 0.98	0.27 0.62 0.88	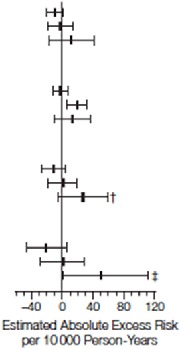	0.22 0.36 0.82	0.17 0.36 0.58	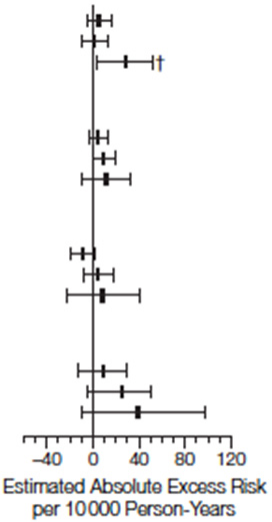
Storke Age Group, y 50–59 60–69 70–79	0.15 0.41 0.70	0.13 0.27 0.55		0.15 0.51 0.76	0.17 0.31 0.59		0.15 0.34 0.63	0.10 0.24 0.52	
Total Mortality Age Group, y 50–59 60–69 70–79	0.24 0.46 1.28	0.34 0.60 1.14		0.28 0.77 1.53	0.38 0.75 1.27		0.21 0.51 1.06	0.30 0.47 1.01	
Global Index Age Group, y 50–59 60–69 70–79	0.97 1.95 3.50	1.01 1.83 3.06		0.95 2.07 3.67	1.15 2.07 3.13		0.99 1.85 3.35	0.89 1.64 2.99	

The estimated absolute excess risk may differ slightly from the absolute excess risk derived from the differences in cases pler 100 person-years betwee active hormone and placebo groups. Estimated absolute exvess risk was per 10 000 person-years calculated as [annualized percentage in the placebo in the palcebo group × (hazard tatio in the placbo group –1)] × 1000. Error bars indicate 95% confidence intervals, estimated using bootstrap methods. CEE indicates conjugated equine estrogens; CHD, coronary heart disease; MPA, medroxyprogesterone acetate. * *P*=.03 compared with the age group of 50 to 59 years. † *P*=.02 compared with the age group of 50 to 59 years. ‡ *P*=.01 compared with the age group of 50 to 59 years.

Although most of the information regarding the possible adverse effects of medroxyprogesterone compared with progesterone deals with breast cancer, there are other aspects that may affect the cardioprotective effect of estrogen ^48, 49^. For example, in the WHI Coronary Artery Calcification Study (WHI-CACS), which lasted a mean of 7.4 years, women in the 50 to 59 age group who received estrogen had lower mean CAC scores than women in the same age group who were given placebo (83.1 versus 123.1, *P* = 0.02). The lack of a protective effect of added MPA is evidence of the cardioprotective effect of estrogen when initiated close to the time of menopause and used without progestogen ^50^.

The most recent evaluation of the WHI results shows that in women in the 50 to 54 age group, who had undergone premature menopause and were treated with ET, all categories of measurement of CVD and of cardiovascular events demonstrated an excess in the placebo group compared with the ET subjects.Unfortunately, for most of the categories, there was insufficient numerical power to prove statistical significance ^23^. A similar result was found by comparing the WHI sub-groups with the observational NHS ^25^.

In two studies of CAC, after prolonged use of hormone replacement therapy, less CAC was found in the users than in never-users ^32, 51^.

The final measure of cardioprotection is evidence of differences in cardiovascular death rates from MHT versus no treatment or placebo. At present, the WHI combined follow-up studies are at 18 years. Although they show a slight lowering in the average number of cardiovascular deaths in timely (starting at 50 to 59 years of age) MHT-treated women, the total number of deaths in that group is too low (18) to furnish statistical significance ^52^. Moreover, the evaluations are bound by the type of regimen used, which is a serious stricture ^42^.

## The timing hypothesis of estrogen cardioprotection may explain the failure of late-start MHT/HT to protect against development of clinical atherosclerosis in the WHI

**Figure 2.**
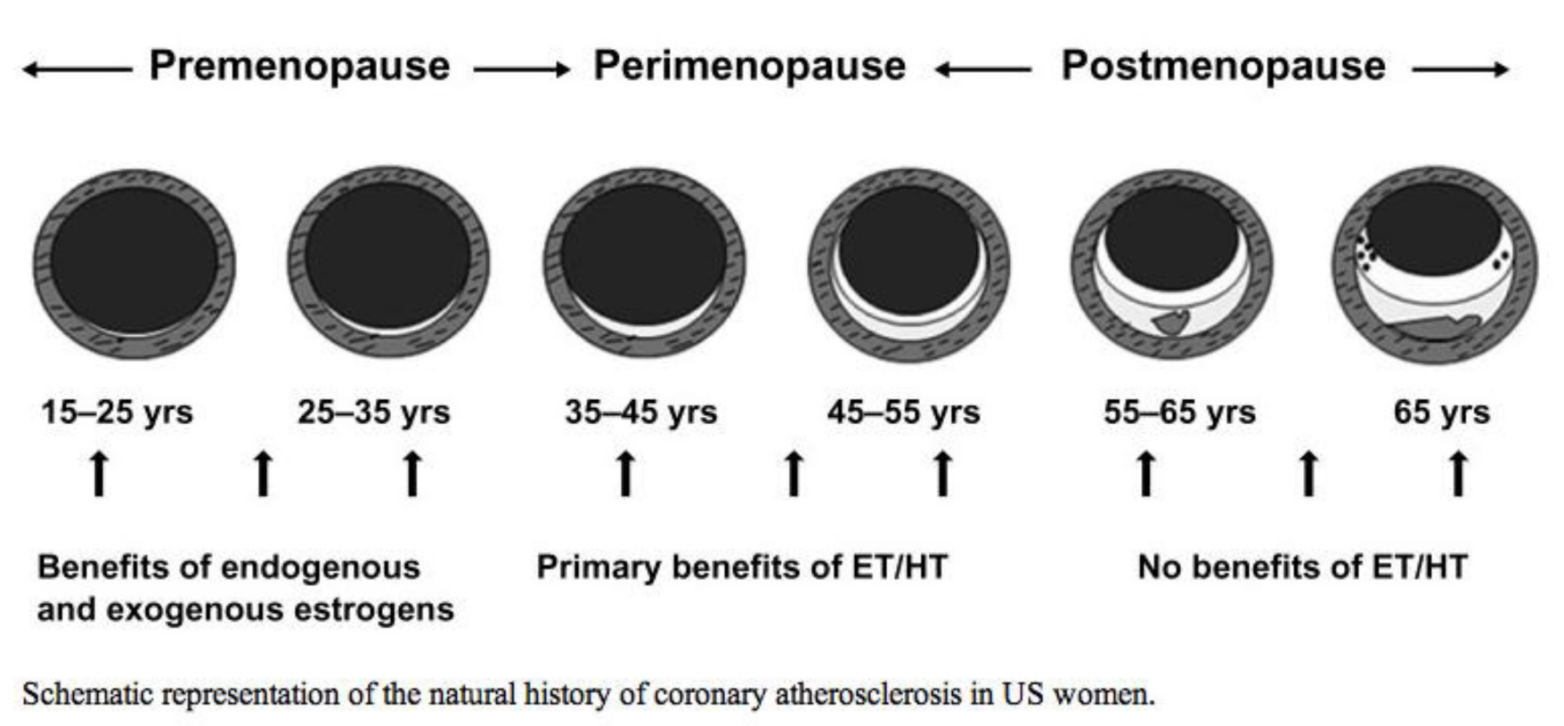
Schematic representation of the natural history of coronary atherosclerosis in American women. ET/HT, estrogen treatment/hormone treatment. Reprinted from Mikkola *et al*. ^53^ with permission.

The timing hypothesis of estrogen-induced cardioprotection first proposed by Thomas Clarkson in 1998 appears to explain the failure of the WHI to show a protective effect of MHT/HT in any but its 50- to 59-year-old subjects. Based on his studies of female cynomolgus monkeys, Clarkson proposed that since atherosclerotic vascular plaque is irreversible by MHT, the beneficial effects of MHT are dependent on initiation before plaque has formed. That the development of plaque is also a product of risk factors was evidenced by his use of a high-lipid diet as the atherogenic stimulus during experiments inducing plaque versus reducing plaque with CEE ^50^. For the sake of brevity, the normal course of plaque development is shown in Figure 2 ^53^. Monocytes are captured by inflamed vascular endothelial cells in part via tethers of nCAM:nCAM molecules expressed by monocytes and endothelial cells ^20^. Coronary vessel endothelial cells express aromatase and estrogen receptors ^13^. Estrogen prevents atherogenesis by inducing sialylases that sialylate (sugar-coat) nCAM molecule’s extracellular domains, thereby blocking the nCAM:nCAM tethers ^13^. Clarkson mimicked these stages by removing the ovaries in female monkeys and then using CEE plus medroxyprogesterone treatment to block plaque formation during feeding with an atherogenic diet. Clarkson found that, once established, estrogen does not remove plaque; when he treated monkeys with CEE after the plaque was established, there was no regression of plaque. He then proposed the timing hypothesis: early ET prevents plaque formation in normal post-menopausal subjects but late treatment does not and therefore the latter is not cardioprotective ^54^.

Many authors have voiced support for the timing hypothesis as an explanation for the failure of the initial interpretation of the WHI cohort to show cardioprotection by ET, and they raised the question of whether the overall rate of VTE in the WHI was related to the presence of sub-clinical atherosclerosis and increased clotting propensity ^55– 57^.

## Confirmation of the timing hypothesis in humans

**Figure 3.**
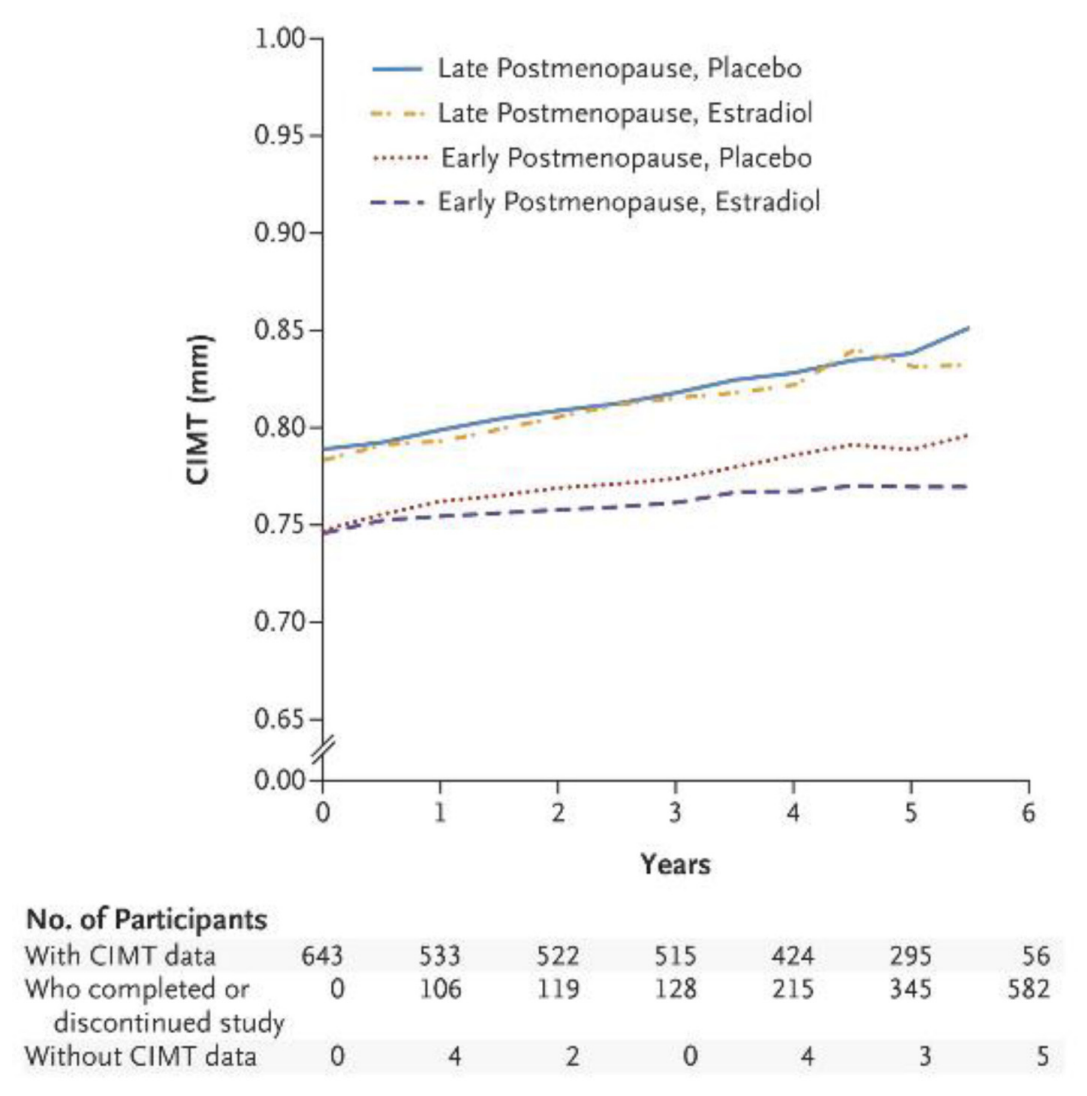
Carotid intima-media thickness (CIMT) progression according to post-menopausal years at the start of treatment. The strata are early (up to 6 years before menopausal hormone treatment, or MHT) and late (>6 years before MHT). Reprinted from Hodis *et al*. ^58^ with permission.

The monkey atheroprotection timing hypothesis was verified in a prospective randomized clinical trial performed explicitly for that purpose. In March 2016, the ELITE Research Group published the first randomized control trial to test the timing hypothesis ^58^. They divided 643 healthy post-menopausal women according to the time since menopause (<6 years or ≥10 years) and randomly assigned them to receive either oral estradiol plus vaginal progesterone or placebo. Their primary outcome was the rate of change in CIMT. They found that, in the early post-menopausal cohort, the rate of CIMT progression was significantly lower in the estradiol group than in the placebo group; this effect was not seen in the late post-menopausal cohort ( Figure 3). Comparable data gleaned from the Peking study ^32^ and the WHI support the findings of the ELITE study ^58^.

Taken together, the multitude of epidemiological, retrospective, and prospective studies support a protective role for properly timed MHT/ET against CVD. Sub-analysis of the WHI plus its ancillary studies strongly supports the cardioprotective effect of ET in menopausal women ^44^. The active agent appears to be the estrogen since the addition of progestin reduces the protective effect. However, it must remain clear that, thus far, only indicators of cardioprotection have been adduced. A prospective study is needed to clarify whether properly timed MHT/ET prevents or delays actual cardiac events such as myocardial infarction, heart failure, or death.

## When to begin menopausal hormone treatment

It is now indisputable that the prevention of CVD depends on the timing of initiation of MHT. *Unless there is a clear contraindication, premature menopause patients (surgical or otherwise) should start treatment at the first opportunity.*

In regard to natural menopause, most governing bodies have recommended the initiation of MHT within the first 10 years of menopause or at the onset of menopausal symptoms ^59, 60^. Hodis *et al*. ^58^ focus on the “menopausal age” over the chronological age. We agree that, when possible, MHT should be initiated as close as possible to menopause or at the onset of menopausal symptoms. Given currently available data comparing the risk of VTE with the cardiovascular benefits of slowing CIMT, 6 years is an appropriate “soft” cutoff for starting MHT in women without high-risk factors for CVD. In the presence of risk factors (hypertension, elevated lipids, and metabolic syndrome), a work-up for clotting factors and sub-clinical CVD is appropriate before starting long-term MHT for any reason. Initiating MHT during this optimal window also allows the opportunity to improve menopausal symptoms, prevent osteoporosis, and treat the metabolic syndrome and its complications ^31^.

Pre-menopausal treatment with MHT is inappropriate because of the large and unpredictable swings of hormones during that period; the most efficient treatments of bleeding and symptoms in properly screened pre-menopausal women are oral contraceptives ^61^.

## In summary

Preclinical and clinical studies support the cardioprotective value of timely (that is, within 5 to 10 years of menopause) application of MHT. This rule appears to hold regardless of the severity of menopausal symptoms. The risk of VTE is related to age and is not a contraindication in properly screened, early menopausal women.

## What treatment to use

This question itself requires its own review and cannot be further addressed here. The data presented in this article address the outcomes of type 1 trials (randomized, blinded, placebo-controlled), except as noted. There are many other treatment regimens that either are not relevant to the specific question of cardiovascular health or were not included because of the paucity of type 1 trial data. To peruse this important subject, we suggest that the reader begin with the current American College of Obstetricians and Gynecologists Practice Bulletin ^59^.

## When to halt treatment

One of the most important lessons to be learned, or re-learned, from the extended evaluation and observational studies of the WHI is confirmation of the positive effects of ET/MHT on symptoms, bone health, metabolic syndrome, and cardiovascular health. This is especially true regarding long-term outcomes ^33, 35, 41, 42^. The follow-up WHI studies regarding cardiovascular events, especially deaths from myocardial infarction or post-infarctional heart failure, are currently reaching sufficient cases to confirm the cardioprotective effects of MHT/ET, but there is no scientifically gathered information about when to halt treatment. There are no prospective data regarding shortening the safe length of treatment or prevention of menopausal complications. On the contrary, there is much data indicating that women can safely receive MHT for extended periods ^7, 32^.

**Figure 4.**
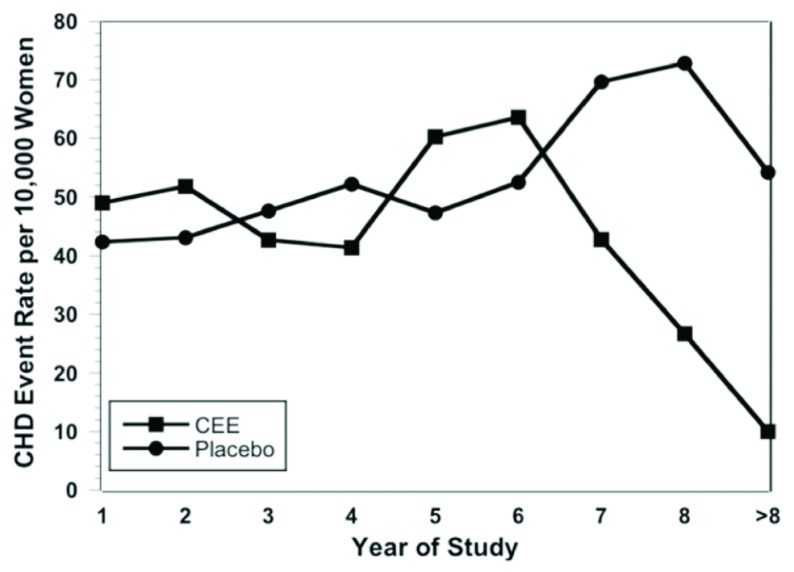
Annual incidence of coronary heart disease events per 10,000 women in the Women’s Health Initiative Estrogen Alone trial. CEE, conjugated equine estrogen; CHD, coronary heart disease. Reprinted from Harman *et al*. ^66^ with permission.

The US Food and Drug Administration recommends that hormones should be used at the lowest dose for the shortest duration consistent with therapeutic goals ^62^, generally taken to mean approximately 5 years maximum. But observational studies evaluating MHT duration suggest that the protective effects of MHT against coronary heart disease may become apparent only after many years of treatment ^63, 64^. In a re-evaluation of the WHI estrogen-plus-progestin arm, Toh *et al*. ^65^ found that women starting MHT within 10 years after menopause had a CVD hazard ratio over the first 2 years of treatment of 1.29 (CI 0.52–3.18) but that after 8 years of treatment this decreased to 0.64 (CI 0.21–1.99). Harman *et al*. ^66^ evaluated published results of the WHI ET trial and found that coronary heart disease incidence rates were similar between placebo and ET patients in years 1 through 6. However, with more than 6 years of ET, there was a statistically significant decrease in CVD risk as compared with the placebo group ( Figure 4). There is ample evidence that closely supervised women taking MHT can safely continue for decades.

The Kronos Early Estrogen Prevention Study (KEEPS) low-dose MHT trial, though showing improvement of risk factors for CVD, failed to show a slowing of the increase of CIMT by 4 years of estradiol-plus-progesterone treatment. This may have been due to the inability of CIMT measurements to reveal cardioprotection that occurred during the 4 years of the trial ^67^ since the ELITE study confirmed that even normal CIMT women have increases in their CIMT after menopause. Under the ELITE hormonal regimen, improved CIMT over placebo treatment was not apparent until 3 or 4 years of treatment and did not reach statistical significance until 5 years of treatment ^58^ ( Figure 3). We believe that the reason for the lack of similar findings in the KEEPS was the exclusion of test subjects who had evidence of sub-clinical CVD (CAC of ≤ 50 Agatston units) at the time of registration ^67^.

At this point, there are no published prospective studies regarding the limits of estrogen-induced cardioprotection in women starting MHT within 10 years of menopause. However, continued bone protection and metabolic protection have been documented ^68^.

With increasing age, issues regarding cognition and general brain deterioration arise. This may proceed to dementia. At present, there is no clinical testing that satisfactorily determines the type of dementia: vascular dementia (VD) or degenerative dementia (Alzheimer’s disease, or AD). In general, both diseases are present in all patients with dementia but the relative impact of each is not known, nor is the effect of age versus other causes, such as estrogen deficiency. Many studies have shown that estrogen may be preventive against aspects of AD and VD. At present, since amyloid deposition is found in brain vessels and nervous system plaques, we must be very guarded about the possible effects of hormones in preventing or treating specific forms of dementia ^69^. Although protection against dementia has been inferred from retrospective studies, the recent mortality rates from the 18-year follow-up of the WHI indicated a reduced occurrence at autopsy of both dementia and AD in the CEE group ^52^. One retrospective report of women who used low dosing regimens for up to 31 years showed no difference in VTE or other adverse events, but the CIMTs and CACs were significantly lower in the patients who received treatment versus controls ^33^.

The effect of age versus MHT on brain VTE remains an unresolved issue. Systemic VTE is a rare and symptomatic occurrence during the first decade after menopause. Brain vessel VTE rates and timing are only known when associated with symptoms, yet the brain, especially the white matter, shrinks with aging. This is under study ^70^.

## In summary

Simply stopping MHT/ET at some arbitrary time seems premature. The length of treatment should be based on health surveillance. This means that MHT administration must be accompanied by regular, periodic examinations, including pelvic exams, cervical cancer testing (especially when there is a change in partners without knowledge of human papillomavirus status), and mammograms. These visits should include discussions with the patient regarding the effects of continuing or halting treatment. For example, MHT protection against the metabolic syndrome and its inflammatory effects is well established, as is protection against bone loss and fracture. Although ET is clearly protective against invasive breast cancer, the jury is still out on ovarian cancer ^71^ and both are often found in post-menopausal women regardless of their history of MHT. The overlap may complicate the discussion but is itself not a reason for halting MHT/ET.

In the case of estrogen and cardioprotection, although trials of more than 5 years have shown the protective effects of MHT (specifically estrogen) up to 11 years and case control studies show protection into the fourth decade of use, there are no trials addressing the acceptable length of treatment. However, women with (predominantly surgical) premature menopause commonly continue estrogen until death, usually decades later and from a variety of causes ^7^.

## A holistic template for prevention and treatment which may afford cardioprotection for post-menopausal women

**Figure 5.**
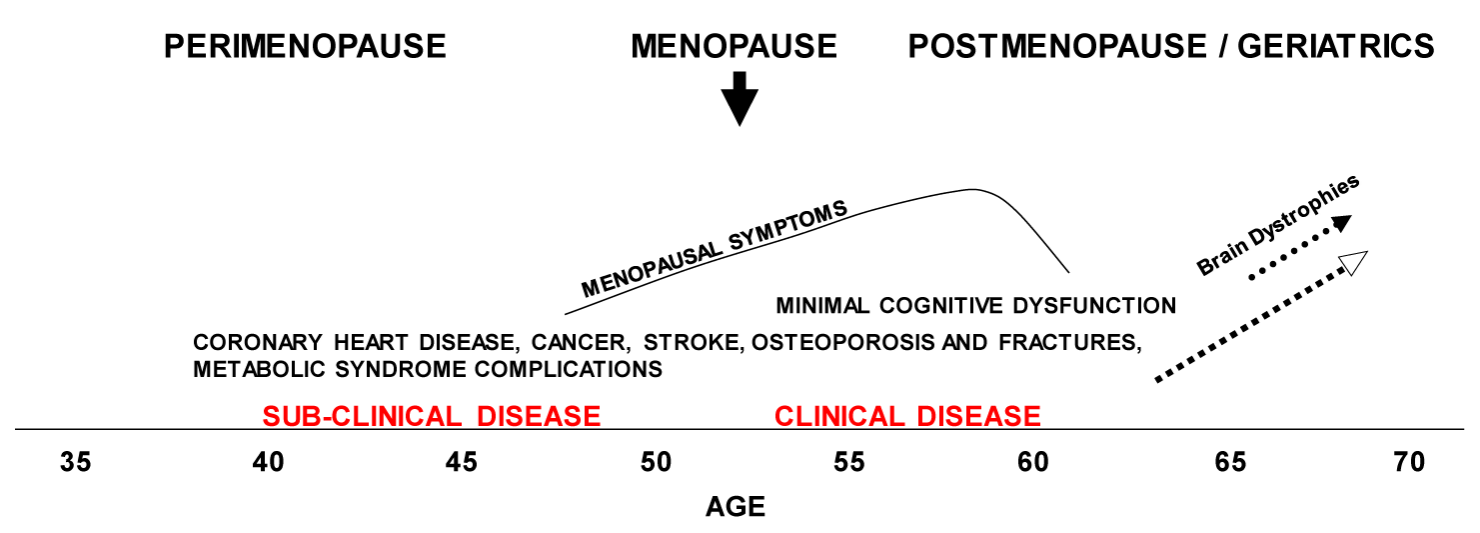
A diagram of the relationship of chronological age and reproductive stage to the development of chronic diseases in women who are vulnerable because of risk factors and lifestyle. Space constraints limit the diseases that may begin or manifest with age. Not all women will undergo these conditions, nor do they all manifest precisely in the periods bracketed.

No woman comes to menopause without the imprint of her genome, intervening events since birth, and environmental/lifestyle effects. This often results in sub-clinical disease prior to the milestone of menopause. If the progress of these ailments is clinically evident, women with sub-clinical CVD may already be receiving care when they cease ovarian estrogen secretion. However, their sub-clinical CVD often will not have been diagnosed or treated. In such cases, the onset of menopausal symptoms can be a defining moment since this brings the woman to the attention of health-care professionals. At that moment, the patient is open to consideration and even acceptance of changes in lifestyle and other aspects of prevention/disease management. Although this is opportune, it is often too late to forestall sub-clinical disease. Figure 5 diagrams the chronological relationship of disease development (including CVD in high-risk women), the chronological age, and reproductive stage. The presence of sub-clinical CVD and endothelial dysfunction predisposes the patient who receives MHT to VTE (see above) and should be carefully evaluated before suggesting ET/MHT. It may be useful to evaluate CIMT and CAC or to perform tests of endothelial dysfunction prior to starting ET/MHT. This is especially true for women who are more than 5 years post-menopause (that is, 6 years since their last menstrual period) ^58^.

**Figure 6.**
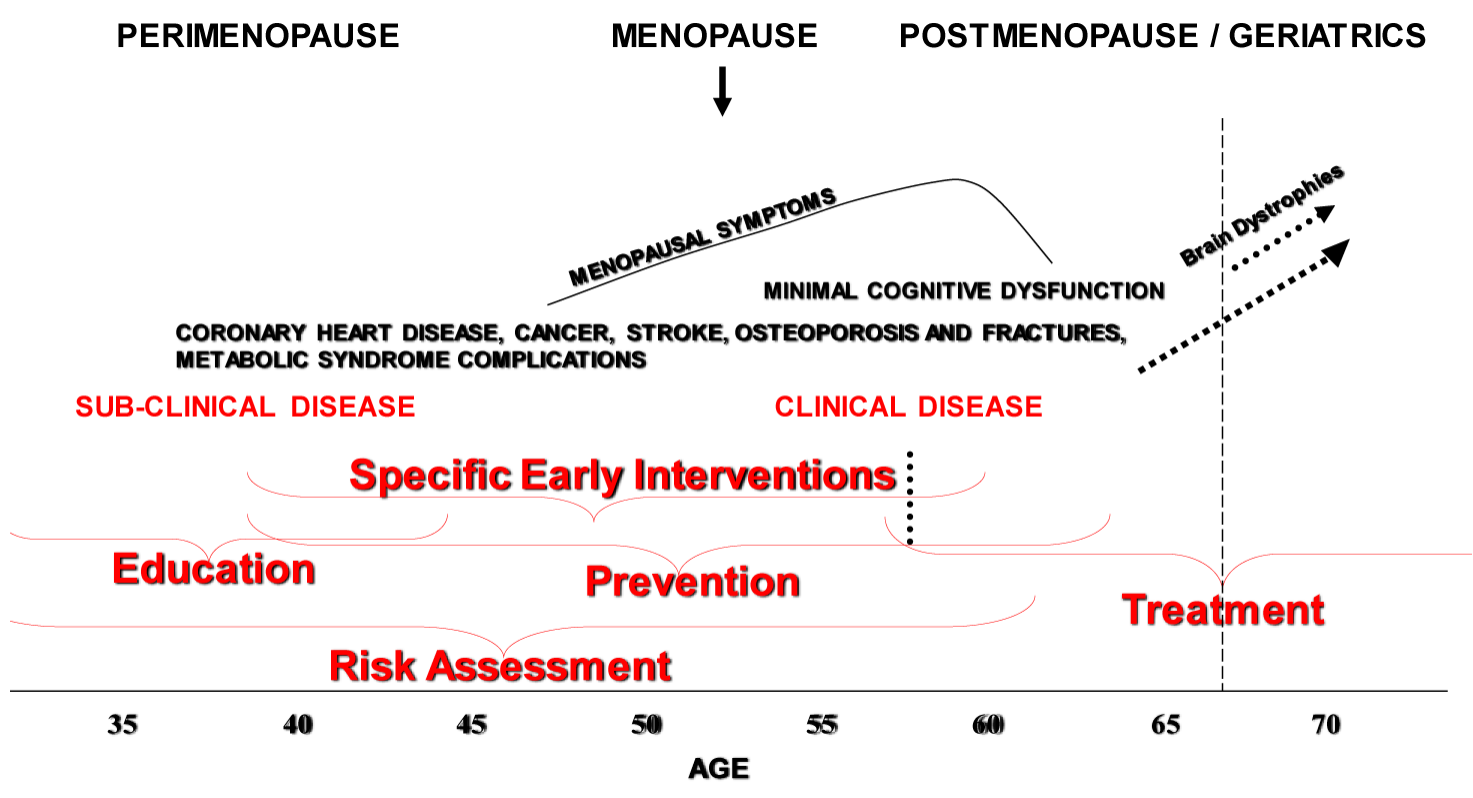
Insertion of the periods in which education, risk assessment, and various interventions, including menopausal hormone treatment (MHT), may be effectively practiced in the aging woman. Annotated version of Figure 5 with additions in red indicating the periods in which education, risk assessment and various interventions, including MHT, may be effectively be practiced in the aging woman. The vertical dotted line approximates the last age of normal women thus far studied for long-term treatment outcomes (see text). The use of treatment into the post-menopause/geriatric period must be accompanied by annual evaluation. Because of the appearance of sub-clinical and clinical disease in the later years, best Practice indicates that this annual visit should not be discontinued after hormone treatment is stopped.

The above descriptions of the genesis of atherosclerosis and the background of the timing hypothesis indicate that starting prevention or treatment of CVD should not await menopause. With this in mind, a program of early education, risk assessment, healthy interventions, and treatment of nascent or sub-clinical disease may be applied. This is a form of preventive medicine that is familiar to primary care practitioners. It does not require high-tech or extreme measures. The largest impediment to employing these measures is the lack of the patient’s openness to evaluation and to lifestyle management. However, life events such as pregnancy, illnesses, and menopause often will open women’s minds to education and prevention. Practitioners should be aware of the significance of these openings and institute appropriate discussions and measures. Figure 6 diagrams in broad strokes the usual periods when risk evaluation, education, and preventative measures may be successfully introduced. These are the times of inflection points in women’s lives, when they are open to reconsideration of lifestyle changes and so on. Regardless of the diagnostic and treatment plan, the key is to thoroughly discuss it with the patient and to in-build periodic (annual) history-taking and diagnostic measures. It goes without saying that the annual visit will also furnish the benefits of timely diagnosis of non-hormone-related disease.

## Summary

In proven menopausal-normal women, timely initiation of MHT for symptoms is safe compared to the menopausal complications that it treats or prevents. Thus far, evidence indicates that MPA is to be avoided because of its possible link to breast cancer ^47^. Although a head to head trial has not been published, our choice is to use MHT cyclically rather than continuously. The evidence adduced thus far supports the cardioprotective effects of MHT. Estrogen alone (or treatment that avoids progestin) is especially convincing. But, the final confirmation awaits proof regarding prevention of actual cardiac events. Since MHT is usually administered for menopausal symptoms and vaginal atrophy/sexual dysfunction, but also deterioration of skin turgor or bone health and cardioprotective effects of ET-MHT, given that the latter are not FDA approved indications for MHT, the decision about whether to start or stop MHT is often made in the absence of clinical research results.

The work-up for treatment of menopausal symptoms in women with CVD risk factors should include CIMT or CAC. An age- and sex-adjusted CIMT measurement may not be necessary for evaluation ^72^. Coronary calcium score is readily available and correlates well with coronary disease. MHT should be combined with lifestyle modification and anti-atherosclerosis medications and regimens. All patients receiving MHT/ET should have annual visits with CVD evaluation.

The decision to continue or stop treatment should be shared between the health-care provider and patient. For most women, the benefits of hormone therapy, including cardioprotection will outweigh the risk. At present, there are no studies indicating a time limit on the administration of properly monitored MHT/ET.

## More research is needed

Unfortunately, the impact of the original report of the WHI caused a massive decline in the use of MHT ^73^. This has resulted in a generally decreased interest in the treatment of menopausal women by all sectors of health care. This includes the pharmaceutical industry, which formerly sponsored many of the clinical trials on MHT. But even had the turning away from the regimens tested been warranted, the problem of management of this important and growing group has not lessened. Rather, it continues to increase as the population ages. Without continuing efforts at improving the drug regimens and removing harmful agents and practices from menopausal health care, it is inevitable that there will be increased aggregate misery, cost, and societal impact of age-related ovarian failure. The findings discussed above should be a call to action to further the search for effective lifestyle regimens, educational programs, diagnostic methods, and hormonal and non-hormonal agents with which to confront not only the increased rate of CVD in the post-menopausal years. There is much more research that can be done to properly innovate and test on behalf of these women.
